# Recent Advances in the Structural Biology of the Volume-Regulated Anion Channel LRRC8

**DOI:** 10.3389/fphar.2022.896532

**Published:** 2022-05-11

**Authors:** Go Kasuya, Osamu Nureki

**Affiliations:** ^1^ Division of Integrative Physiology, Department of Physiology, Jichi Medical University, Shimotsuke, Japan; ^2^ Department of Biological Sciences, Graduate School of Science, The University of Tokyo, Tokyo, Japan

**Keywords:** structural biology, cryo-EM, cell volume homeostasis, VRAC, LRRC8

## Abstract

Members of the leucine-rich repeat-containing 8 (LRRC8) protein family, composed of five LRRC8A-E isoforms, are pore-forming components of the volume-regulated anion channel (VRAC), which is activated by cell swelling and releases chloride ions (Cl^−^) or other osmolytes to counteract cell swelling. Although the LRRC8 protein family was identified as the molecular entity of VRAC only in 2014, due to recent advances in cryo-electron microscopy (cryo-EM), various LRRC8 structures, including homo-hexameric LRRC8A and LRRC8D structures, as well as inhibitor-bound and synthetic single-domain antibody-bound homo-hexameric LRRC8A structures, have been reported, thus extending our understanding of the molecular mechanisms of this protein family. In this review, we describe the important features of LRRC8 provided by these structures, particularly the overall architectures, and the suggested mechanisms underlying pore inhibition and allosteric modulation by targeting the intracellular leucine-rich repeat (LRR) domain.

## Introduction

Maintenance of cell volume is a fundamental process for proper cell functions. Various membrane channels and transporters that are activated by cell swelling or cell shrinkage are associated with this process (Hoffmann et al., 2009). Among them, the volume-regulated anion channel (VRAC) is a particular type of anion channel (Jentsch, 2016). VRAC is activated by cell swelling and mediates the transport of Cl^−^ and small organic compounds that can serve as osmolytes. The VRAC-mediated transport of ions and osmolytes leads to water efflux and thereby returns the cell volume back to normal, a process that is called regulatory volume decrease (RVD) (Okada et al., 2019). In various eukaryotic cells including HEK293 cells, HeLa cells, and HCT116 cells, the VRAC-mediated currents are inhibited by the VRAC inhibitor DCPIB {4-[(2-Butyl-6,7-dichloro-2-cyclopentyl-2,3-dihydro-1-oxo-1H-inden-5-yl)oxy]butanoic acid} (Decher et al., 2001; Sato-Numata et al., 2016; Yamada et al., 2021). The existence of VRAC in human T lymphocytes and human intestinal epithelial cells was proposed in the 1980s (Nilius et al., 1997). Subsequent studies revealed various biophysical characteristics of VRAC, including high anion selectivity, weak outward rectification, permeability to organic osmolytes, and modulation by intracellular ATP (Pedersen et al., 2016; König and Stauber, 2019). However, despite extensive studies over the past decades, the molecular entity of VRAC had not been identified until recently mainly due to its ubiquitous expression and complex biophysical properties.

In 2014, using the high-throughput siRNA screening strategy combined with a halide-sensitive YFP, two independent groups found that leucine-rich repeat-containing 8A (LRRC8A), a mutation in which was found in a patient lacking B cells in peripheral blood (Sawada et al., 2003), is an essential component of VRAC (Qiu et al., 2014; Voss et al., 2014). LRRC8A is a member of the LRRC8 protein family composed of five LRRC8A-E isoforms. The LRRC8 protein family was thought to have a membrane topology similar to that of pannexin, connexin, and innexin (Abascal and Zardoya, 2012). These channels are permeable to small ions (e.g., Na^+^, K^+^, Ca^2+^, and Cl^−^) as well as larger molecules (e.g., ATP) (Ma et al., 2016; Chiu et al., 2018), and it has recently proposed that they should be classified as large-pore channels (Syrjanen et al., 2021) [Structural and functional comparisons between LRRC8 and other large-pore channels are well-summarized in a recent review (Syrjanen et al., 2021).] Hetero-hexameric assembly of LRRC8A and at least one other LRRC8 isoform is needed for the formation of a functional VRAC under physiological conditions (Voss et al., 2014; Syeda et al., 2016). The combination of LRRC8A and other LRRC8 isoforms determines the biophysical properties of VRAC including open probability (Syeda et al., 2016), gating kinetics (Voss et al., 2014; Ullrich et al., 2016), and substrate specificity (Planells-Cases et al., 2015; Gaitán-Peñas et al., 2016; Lutter et al., 2017; Schober et al., 2017; Lahey et al., 2020). In contrast, the LRRC8A homo-hexamer still retains channel activity in *LRRC8^−/−^* HEK293 cells (Deneka et al., 2018; Yamada et al., 2021) and in lipid-embedded conditions (Syeda et al., 2016; Kasuya et al., 2018), although the LRRC8A homomer is not observed under physiological conditions.

Identification of LRRC8 as the molecular entity of VRAC has also accelerated our understanding of the physiological and medical aspects of the LRRC8-mediated VRAC current (Osei-Owusu et al., 2018; Chen et al., 2019; Figueroa and Denton, 2022). For example, the LRRC8-mediated VRAC current is implicated in adipocyte development (Zhang et al., 2017), insulin secretion (Zhang et al., 2017; Kang et al., 2018; Stuhlmann et al., 2018; Gunasekar et al., 2022), and sperm development (Bao et al., 2018; Lück et al., 2018). VRAC in hippocampal astrocytes transports glutamates to regulate synaptic transmission and neuronal excitability (Yang et al., 2019). VRAC containing the LRRC8D isoform transports platinum-containing drugs, such as cisplatin and carboplatin, and thus is implicated in tumor drug resistance (Planells-Cases et al., 2015). VRAC containing LRRC8C and/or LRRC8E isoforms in macrophages transports an immunotransmitter, 2′3′cyclic-GMP-AMP (cGAMP), to regulate the stimulator of interferon genes (STING) pathway and the production of type I interferons (Lahey et al., 2020; Zhou et al., 2020). VRAC containing the LRRC8C isoform expressed in T cells transports cGAMP to regulate activation of the STING-p53 signaling pathway (Concepcion et al., 2022). These findings suggest that the LRRC8 protein family can be a potential therapeutic target for various diseases such as obesity, diabetes, and stroke.

The emergence of single-particle cryo-electron microscopy (cryo-EM) as a powerful tool for the structural determination of proteins had a great impact on the field of structural biology (Cao et al., 2013; Liao et al., 2013; Cheng, 2018). Due to recent advances in cryo-EM, the first structure of LRRC8 was reported in 2018, only 4 years after the identification of LRRC8 as the molecular entity of VRAC (Deneka et al., 2018). Since then, six studies reported the LRRC8 structures to date (Kasuya et al., 2018; Kefauver et al., 2018; Kern et al., 2019; Nakamura et al., 2020; Deneka et al., 2021; Gunasekar et al., 2022). In this review, we describe the structural and functional features of the LRRC8 protein family that were elucidated by those studies and discuss the possible directions of further research in this field.

### Structure Determination

**FIGURE 1:**
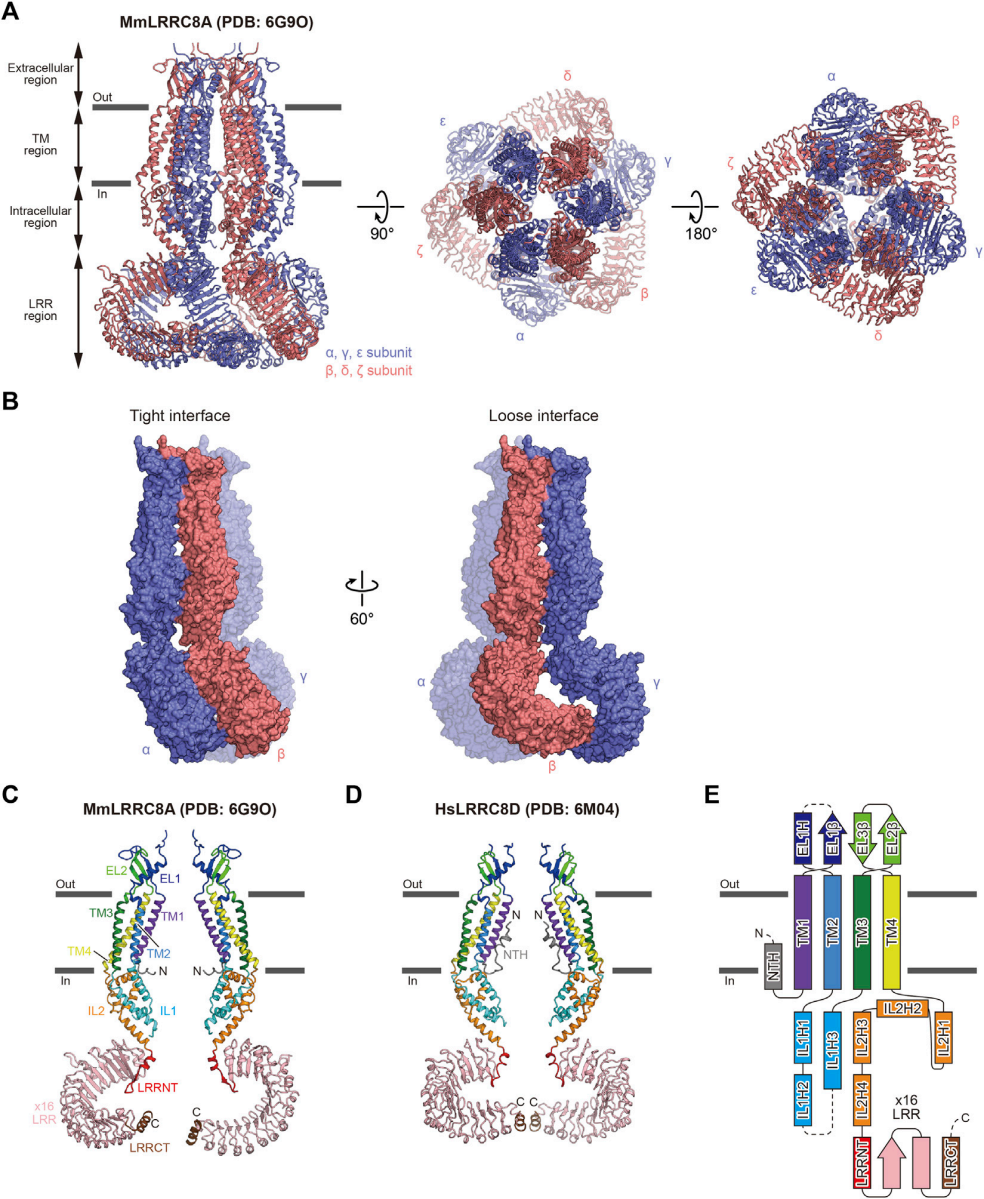
Overall structure and subunit interactions of LRRC8. **(A)** Overall structure of the MmLRRC8A hexamer in detergent (PDB: 6G9O) (Deneka et al., 2018), viewed parallel to the membrane (left) and from the extracellular (middle) and intracellular (right) sides **(B)** Tight (left) and loose (right) subunit interfaces between the three neighboring subunits of the MmLRRC8A hexamer in detergent (PDB: 6G9O) (Deneka et al., 2018) **(C,D)** Two diagonal subunits of the MmLRRC8A hexamer in detergent (PDB: 6G9O) (Deneka et al., 2018) **(C)** and the HsLRRC8D hexamer in detergent (PDB: 6M04) (Nakamura et al., 2020) **(D)**. According to the previously proposed nomenclature (Kasuya et al., 2018; Nakamura et al., 2020), each region is colored as follows: NTH, N-terminal helix, gray; TM1, purple; EL1, blue; TM2, light blue; IL1, cyan; TM3, green; EL2, light green; TM4, yellow; IL2, orange; LRRNT, leucine-rich repeat N-terminal, red; LRR1–16, pink; and LRRCT, brown. The N and C termini are indicated by ‘N’ and ‘C’, respectively **(E)** Schematic representation of the LRRC8 membrane topology, colored as in **(C,D)**. The dotted lines indicate the disordered linkers. Figures prepared with CueMol (http://www.cuemol.org/).

In 2018, the first overall structures of the LRRC8 protein family, mouse LRRC8A homo-hexamer (MmLRRC8A; “Mm” referring to *Mus musculus*) in detergent and low-resolution MmLRRC8A/8C hetero-hexamer in detergent, were determined by cryo-EM (Deneka et al., 2018) (Figures 1A–C). That paper also showed the intracellular LRR region structure of MmLRRC8A determined by X-ray crystallography (Deneka et al., 2018). Soon after publication of that paper, two overall structures of the human LRRC8A homo-hexamer (HsLRRC8A; “Hs” referring to *Homo sapiens*) in detergents were determined by cryo-EM (Kasuya et al., 2018; Kefauver et al., 2018). These MmLRRC8A and HsLRRC8A homo-hexamer structures revealed the overall architecture, ion permeation pathway, and selectivity filter. In 2019, the transmembrane (TM) region structures of DCPIB-bound and apo MmLRRC8A homo-hexamer in nanodiscs were determined by cryo-EM (Kern et al., 2019). Nanodiscs are self-assembling lipid bilayers that are stabilized by membrane scaffold proteins (MSP) (Ritchie et al., 2009; Autzen et al., 2019). The DCPIB-bound MmLRRC8A homo-hexamer structure revealed the binding sites of DCPIB and provided a possible DCPIB inhibition mechanism. In 2020, the overall structure of HsLRRC8D homo-hexamer in detergents was determined by cryo-EM (Nakamura et al., 2020), revealing the N-terminal helix involved in ion permeation. In 2021, the overall structures of MmLRRC8A homo-hexamer in complex with five different sybodies were determined by cryo-EM (Deneka et al., 2021). Sybodies are synthetic single-domain antibodies that bind to specific regions of target proteins (Zimmermann et al., 2018). These sybody-bound MmLRRC8A structures provided mechanistic insights into the allosteric modulation in the intracellular leucine-rich repeat (LRR) region. In 2022, the TM region structures of MmLRRC8A homo-hexamer in nanodiscs in complex with SN-407, a DCPIB analog, were determined by cryo-EM (Gunasekar et al., 2022). The DCPIB analog-bound MmLRRC8A homo-hexamer structures provided the structural basis for optimizing chemical compounds targeting the channel pore of LRRC8. The structures of LRRC8 that have been reported to date are listed in Table 1.

**TABLE 1:** Structures of LRRC8 reported to date

Structure	Organism	Other molecule	Method	Resolution(Å)	LRR symmetry	PDB ID	EMDB ID	Reference
Full length								
LRRC8A homo-hexamer	Mus musculus	-	Cryo-EM	5.3	C3	6G9L	4366	Deneka et al (2018)
LRRC8A homo-hexamer	Mus musculus	-	Cryo-EM	4.3	C3	6G9O	4367	Deneka et al (2018)
LRRC8A/8C hetero-hexamer	Mus musculus	-	Cryo-EM	7.94(map)	C3	-	4361	Deneka et al (2018)
LRRC8A homo-hexamer	Homo saplens	-	Cryo-EM	4.4	C3	6DJB	7935	Kefauver et al (2018
LRRC8A homo-hexamer	Homo saplens	-	Cryo-EM	4.25	C3	5ZSU	6952	Kasuya et al (2018)
LRRC8D homo-hexamer	Homo saplens	-	Cryo-EM	4.36	C2	6M04	30029	Nakamura et al (2020)
LRRC8A homo-hexamer	Mus musculus	Sybody(Sb1)	Cryo-EM	3.26	C3	7P5V	13202	Deneka et al (2021)
LRRC8A homo-hexamer	Mus musculus	Sybody(Sb2)	Cryo-EM	3.8	C3	7P5W	13203	Deneka et al (2021)
LRRC8A homo-hexamer	Mus musculus	Sybody(Sb3)	Cryo-EM	3.5	C3	7P5Y	13208	Deneka et al (2021)
LRRC8A homo-hexamer	Mus musculus	Sybody(Sb4)	Cryo-EM	7.7(map)	C3	-	13212	Deneka et al (2021)
LRRC8A homo-hexamer	Mus musculus	Sybody(Sb4)	Cryo-EM	3.9	C3	7P60	13213	Deneka et al (2021)
LRRC8A homo-hexamer	Mus musculus	Sybody(Sb5)	Cryo-EM	5.7	C3	7P6K	13230	Deneka et al (2021)
Pore domain								
LRRC8A homo-hexamer	Mus musculus	-	Cryo-EM	3.7	-	6G8Z	4362	Deneka et al (2018)
LRRC8A homo-hexamer(constricted)	Mus musculus	DCPIB	Cryo-EM	3.32	-	6NZW	0562	Kern et al (2019)
LRRC8A homo-hexamer(expanded)	Mus musculus	DCPIB	Cryo-EM	3.47	-	6NZZ	0563	Kern et al (2019)
LRRC8A homo-hexamer(constricted	Mus musculus	-	Cryo-EM	3.8	-	6O00	0564	Kern et al (2019)
LRRC8A homo-hexamer	Mus musculus	SN-407(Pose-1)	Cryo-EM	3.72	-	7M17	23614	Gunasekar et al (2022)
	Mus musculus	SN-407(Pose-2)	Cryo-EM	3.75	-	7M19	23616	Gunasekar et al (2022)
LRR domain								
LRRC8A protomer	Mus musculus	-	X-ray	1.8	-	6FNW	-	Deneka et al (2018)

### Overall Architecture

The overall architecture and subunit folding are the same in all of the LRRC8 structures that have been determined (Deneka et al., 2021, 2018; Gunasekar et al., 2022; Kasuya et al., 2018; Kefauver et al., 2018; Kern et al., 2019; Nakamura et al., 2020). The overall architecture of LRRC8 assembles into a hexamer (Figure 1A). The subunit consists of four regions: extracellular region, TM region, intracellular region, and LRR region (Figures 1C–E). The extracellular, TM, and intracellular regions are in the N-terminal half of the subunit, while the LRR region is in the C-terminal half of the subunit. The extracellular region protrudes above the cell membrane and forms the channel pore with the TM and intracellular regions. The extracellular region consists of two extracellular loops (EL1 and EL2). EL1 possesses one α-helix (EL1H) and one β-strand (EL1β), while EL2 possesses two β-strands (EL2β1 and EL2β2). EL1 is longer and more variable than EL2 among the LRRC8 isoforms. The TM region consists of four transmembrane helices (TM1-4). TM1 and TM2 are connected by EL1 of the extracellular region, and TM2 and TM3 are connected by the intracellular loop (IL1) of the intracellular region. IL2 in the intracellular region connects TM4 and the LRR region. IL1 possesses three α-helices (IL1H1, IL1H2, and IL1H3), while IL2 possesses four α-helices (IL2H1, IL2H2, IL2H3, and IL2H4). The LRR region consists of the leucine-rich repeat N-terminal helix (LRRNT), up to 16 leucine-rich repeats (LRR1-16), and the leucine-rich repeat C-terminal helix (LRRCT). In all determined LRRC8 structures, the EL1 loop between EL1H and EL1β as well as the IL1 loop between IL1H2 and IL1H3 contain disordered regions, suggesting that these loops have conformational flexibility. In the structure of the HsLRRC8D hexamer, an additional N-terminal helix (NTH) formed by the N-terminal residues preceding TM1 is observed (Nakamura et al., 2020) (Figures 1D,E). One thing to note is that in most of the determined LRRC8 structures, while the extracellular and TM regions are sufficiently well resolved to place models, the other regions are not. Another thing to note is that since 3D reconstruction of protein structures by cryo-EM is generally achieved by using a fraction of the collected protein particles, it is difficult to exclude the possibility that the unused fractions contain other biologically important features, such as oligomeric symmetry or stoichiometry. Therefore, the readers need to take account of the resolution of each region and workflow of 3D reconstruction when evaluating the descriptions in each report.

The extracellular, TM, and intracellular regions have six-fold or three-fold (pseudo six-fold) symmetry in the LRRC8A structures (Deneka et al., 2018, Deneka et al., 2021; Kasuya et al., 2018; Kefauver et al., 2018; Kern et al., 2019; Gunasekar et al., 2022) and two-fold (pseudo six-fold) symmetry in the LRRC8D structure (Nakamura et al., 2020). In contrast, the symmetry of the LRR region is variable, suggesting its conformational flexibility. While the LRR region has three-fold symmetry in the structures of MmLRRC8A in detergent (Deneka et al., 2018) and HsLRRC8A in detergent (Kasuya et al., 2018; Kefauver et al., 2018), it has two-fold symmetry in the structure of HsLRRC8D in detergent (Nakamura et al., 2020). The LRR region is not resolved well in the structures of MmLRRC8A in nanodiscs (Kern et al., 2019; Gunasekar et al., 2022). As a result of the structural difference in the LRR region, there are two manners of interactions observed at the interfaces between the subunits, termed “tight” interaction and “loose” interaction (Figure 1B). For example, *α* and *β* subunits form a “tight” interaction, while *β* and *γ* subunits form a “loose” interaction. However, how these differences in interaction manners influence channel function is currently unknown.

### Features for Ion Permeation

**FIGURE 2:**
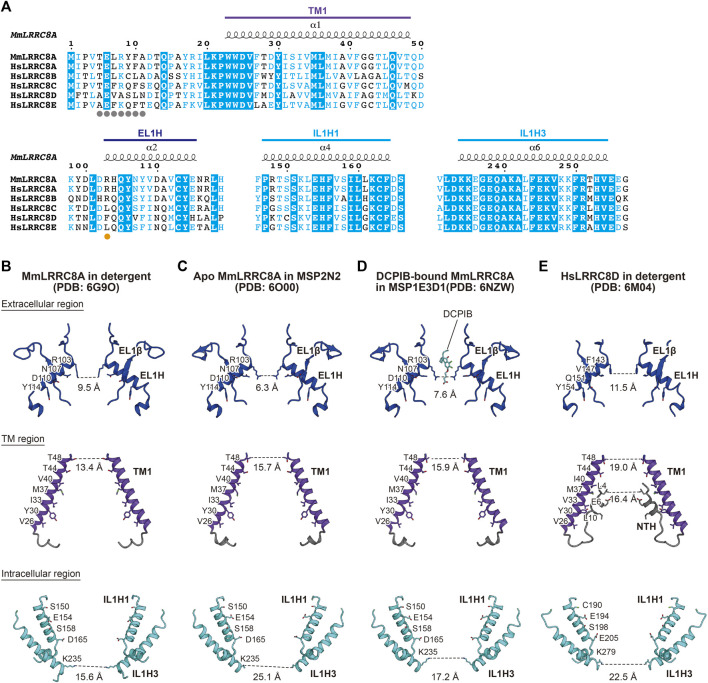
Ion channel pore. **(A)** Sequence alignment around the ion channel pore of LRRC8 isoforms. Amino acid sequences were aligned using Clustal Omega (Sievers et al., 2011) and are shown using ESPript3 (Robert and Gouet, 2014). The secondary structure elements and the colors of the domains from the MmLRRC8A hexamer in detergent (PDB: 6G9O) (Deneka et al., 2018) are labeled above the alignments. The amino acid residue involved in the pore constriction on the extracellular side is highlighted with an orange dot and those involved in the NTH formation in HsLRRC8D (Nakamura et al., 2020) are highlighted with gray dots. For the sequence alignment, the following LRRC8 isoforms were used: mouse (MmLRRC8A, NCBI Reference sequence number: NP_808,393.1) and human (HsLRRC8A, NP_062,540.2; HsLRRC8B, NP_056,165.1; HsLRRC8C, NP_115,646.3; HsLRRC8D, NP_060,573.2; HsLRRC8E, NP_079,337.2) **(B–E)** Close-up views of the channel pore-forming regions of MmLRRC8A in detergent (PDB: 6G9O) (Deneka et al., 2018) **(B)**, apo MmLRRC8A in MSP2N2 nanodiscs (PDB: 6O00) (Kern et al., 2019) **(C)**, DCPIB-bound, constricted MmLRRC8A in MSP1E3D1 nanodiscs (PDB: 6NZW) (Kern et al., 2019) **(D)**, and HsLRRC8D in detergent (PDB: 6M04) (Nakamura et al., 2020) **(E)**. Only two diagonal subunits viewed parallel to the membrane are shown for clarity. The side chains of the pore-lining amino acid residues are depicted in stick representations. The distances between the atoms in PDB coordinates involved in the pore constrictions are shown.

The channel pore of LRRC8 is located along the central axis. It is mainly formed by the EL1H helix in the extracellular region, the TM1 helix in the TM region, and the IL1H1 and IL1H3 helices in the intracellular region (Deneka et al., 2021, Deneka et al., 2018; Kasuya et al., 2018; Kefauver et al., 2018; Kern et al., 2019; Nakamura et al., 2020; Gunasekar et al., 2022) (Figure 2). These residues are mainly hydrophilic and positively charged, enabling anion permeation. A previous electrophysiological study demonstrated that the T44 residue of LRRC8A is involved in ion permeation (Syeda et al., 2016). In the LRRC8A structures, the T44 residue is located on TM1 and faces the channel pore (Deneka et al., 2018, Deneka et al., 2021; Kasuya et al., 2018; Kefauver et al., 2018; Kern et al., 2019; Gunasekar et al., 2022). The diameters of the pore are about 6.3–11.5 Å on the extracellular side, and the pore widens as it goes down to the TM region and then narrows to about 15.6–25.1 Å on the intracellular side (Figures 2B–E). The most constricted site at the extracellular side is formed by the side chains of Arg103 residues in LRRC8A and those of Phe143 residues in LRRC8D. These residues are located at the N-terminal tip of the EL1H helix (Figure 2). As a result of these differences in amino acid residues, the most constricted sites in the LRRC8A homo-hexamer structures are narrower than that in the LRRC8D homo-hexamer structure (Figures 2B–E). Importantly, the R103 residue is only conserved in LRRC8A and LRRC8B among the LRRC8 isoforms (Figure 2A). Although the LRRC8A/8B hetero-hexamer can form a functional VRAC in HCT116 cells, how the LRRC8B isoform is involved in the native VRAC is currently unknown. Therefore, considering that LRRC8A and at least one other LRRC8 isoform assemble into hetero-hexamers, the inclusion of the LRRC8C, LRRC8D, and/or LRRC8E isoforms into VRAC may change the pore diameter and pore polarity at the most constricted site and thereby affect the permeability of VRAC substrates (Figures 2B–E).

In the HsLRRC8D structure, an additional NTH helix is formed by the Ala5 to Asn11 residues at the N-terminal region (Nakamura et al., 2020) (Figures 2A,E). The NTH helix enters the channel pore from the intracellular side and lines the channel pore as in the case of other large-pore channels (Syrjanen et al., 2021). The importance of the N-terminal region is supported by the results of previous electrophysiological studies showing that the N-terminal region affects the biophysical properties of VRAC including conductance, ion permeability, and gating kinetics (Kefauver et al., 2018; Zhou et al., 2018).

It is notable that a recent electrophysiological study in which the functional property of the LRRC8A homo-hexamer was assessed suggested that these LRRC8A homo-hexameric structures should be used with caution as structure-based guides for mutagenesis studies (Yamada et al., 2021). That study showed that while the native VRAC and LRRC8A/8C hetero-hexamer are activated by either cell swelling or low intracellular µ or both but that the LRRC8A homo-hexamer is only activated by cell swelling under the condition of low intracellular µ. These differences suggest that the LRRC8A homo-hexamer forms a conformation with impaired sensing of cell volume or low intracellular µ or both.

### Pore Inhibition

**FIGURE 3:**
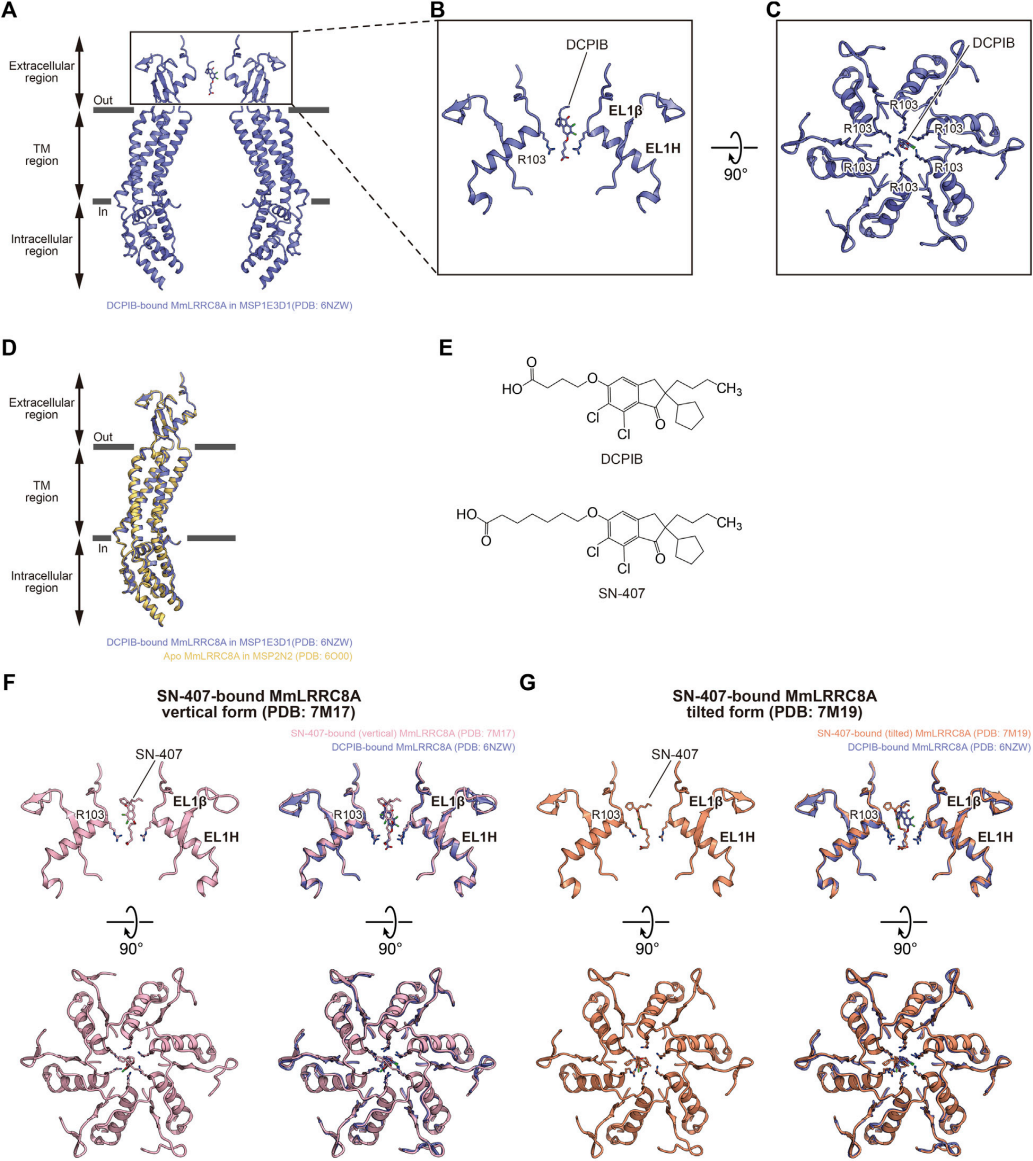
Pore inhibition. **(A–C)** Overall **(A)** and close-up **(B,C)** views of the DCPIB binding site of DCPIB-bound, constricted MmLRRC8A in MSP1E3D1 nanodiscs (PDB: 6NZW) (Kern et al., 2019). The DCPIB molecule and side chains of R103 residues are depicted in stick representations. In **(A,B)** only two diagonal subunits viewed parallel to the membrane are shown for clarity. In **(C)**, all six subunits viewed from the extracellular side are shown. **(D)** Superimposition of DCPIB-bound, constricted MmLRRC8A in MSP1E3D1 nanodiscs (PDB: 6NZW) (Kern et al., 2019) (blue) and apo MmLRRC8A in MSP2N2 nanodiscs (PDB: 6O00) (Kern et al., 2019) (orange), using the Cα atoms (residues Pro15-Asn408) of the subunits. The root mean square deviation (RMSD) value is 0.50 Å for 303 Cα atoms. **(E)** Chemical structures of DCPIB and SN-407. **(F)** Close-up view of the SN-407 binding site of SN-407-bound MmLRRC8A in MSP1E3D1 nanodiscs (vertical form) (PDB: 7M17) (Gunasekar et al., 2022) (left). Superimposition of SN-407-bound MmLRRC8A in MSP1E3D1 nanodiscs (vertical form) (PDB: 7M17) (Gunasekar et al., 2022) and DCPIB-bound, constricted MmLRRC8A in MSP1E3D1 nanodiscs (PDB: 6NZW) (Kern et al., 2019) (right). **(G)** Close-up view of the SN-407 binding site of SN-407-bound MmLRRC8A in MSP1E3D1 nanodiscs (tilted form) (PDB: 7M19) (Gunasekar et al., 2022) (left). Superimposition of SN-407-bound MmLRRC8A in MSP1E3D1 nanodiscs (tilted form) (PDB: 7M19) (Gunasekar et al., 2022) and DCPIB-bound, constricted MmLRRC8A in MSP1E3D1 nanodiscs (PDB: 6NZW) (Kern et al., 2019) (right).

The structure of the DCPIB-bound MmLRRC8A homo-hexamer in nanodiscs revealed the DCPIB binding mode (Kern et al., 2019) (Figures 3A–E). DCPIB is positioned at the most constricted site in the extracellular region and is oriented vertically to the membrane. DCPIB is recognized by the side chains of the positively charged Arg103 residues through its carboxylic acid end. In contrast, the bulky hydrophobic end of DCPIB seems to be too large to pass the most constricted site formed by the side chains of Arg103 residues (Figures 3A–C). A structural comparison between the DCPIB-bound and apo MmLRRC8A homo-hexamers in nanodiscs showed that both structures are superimposed well (Figure 3D). Accordingly, these results suggest that the DCPIB molecule acts as a cork to plug the most constricted site from outside and that the DCPIB binding to the channel pore seems to have little effect on the overall conformation. Very recently determined structures of the MmLRRC8A homo-hexamer in nanodiscs in complex with SN-407 further support the DCPIB-dependent inhibition mechanism suggested by the structure of the DCPIB-bound MmLRRC8A homo-hexamer (Gunasekar et al., 2022). SN-407 is one of the DCPIB analogs that have been designed and synthesized according to the structure of the DCPIB-bound MmLRRC8A homo-hexamer (Kern et al., 2019). Compared to DCPIB, SN-407 has a longer carbon chain between the carboxylic acid and the bulky hydrophobic end, and it functions as a more potent inhibitor for the native VRAC in HEK293 cells (Gunasekar et al., 2022) (Figure 3E). While the SN-407 densities are not clear, there were two binding manners of SN-407, termed “vertical” and “tilted” forms, at the most constricted site in the extracellular region (Figures 3F,G). In the vertical form, SN-407 is oriented vertical to the membrane and is recognized by the side chains of Arg103 residues through its carboxylic acid end, as in the case of DCPIB (Kern et al., 2019) (Figure 3F). In the tilted form, the carboxylic acid end of SN-407 is recognized by the side chains of Arg103 residues, as in the case of the vertical form (Figures 3F,G). However, in addition, the bulky hydrophobic end of SN-407 tilts away from the central axis and is recognized by the hydrophobic interface between two neighboring subunits (Figure 3G). This additional hydrophobic interaction may explain the higher inhibitory activity of SN-407 than that of DCPIB. A recent electrophysiological study showed that the inhibitory activity of DCPIB on the LRRC8A homo-hexamer is much weaker than that on the native VRAC (Yamada et al., 2021), suggesting that the DCPIB binding mode observed in the structure of the DCPIB-bound MmLRRC8A homo-hexamer in nanodiscs may not represent the binding mode in the native VRAC. In addition, while studies using animal models suggested that DCPIB has a potential as a therapeutic agent for the treatment of obesity, diabetes, and stroke (Zhang et al., 2008; Gunasekar et al., 2022), there are significant challenges to overcome. For example, it seems that DCPIB cannot cross the blood-brain barrier (BBB) (Zhang et al., 2008). Moreover, DCPIB is not just a specific VRAC inhibitor. At concentrations used to inhibit VRAC, various studies suggested that it regulates other membrane proteins including glutamate transporter GLT-1 (Bowens et al., 2013), Connexin 43 (Cx43) (Bowens et al., 2013), gastric H^+^,K^+^-ATPase (Fujii et al., 2015), components of mitochondrial electron transport chains (Afzal et al., 2019), K2P K^+^ channels (Minieri et al., 2013; Lv et al., 2019), inward rectifying K^+^ (Kir) channels (Deng et al., 2016), and BK K^+^ channel (Zuccolini et al., 2022). Nevertheless, this successful result of the structure-based approach to design DCPIB analogs suggests that further potent inhibitors targeting the native VRAC pore can be designed using the SN-407- and DCPIB-bound MmLRRC8A homo-hexamer structures in the future.

### Allosteric Modulation by Targeting Leucine-Rich Repeat

**FIGURE 4:**
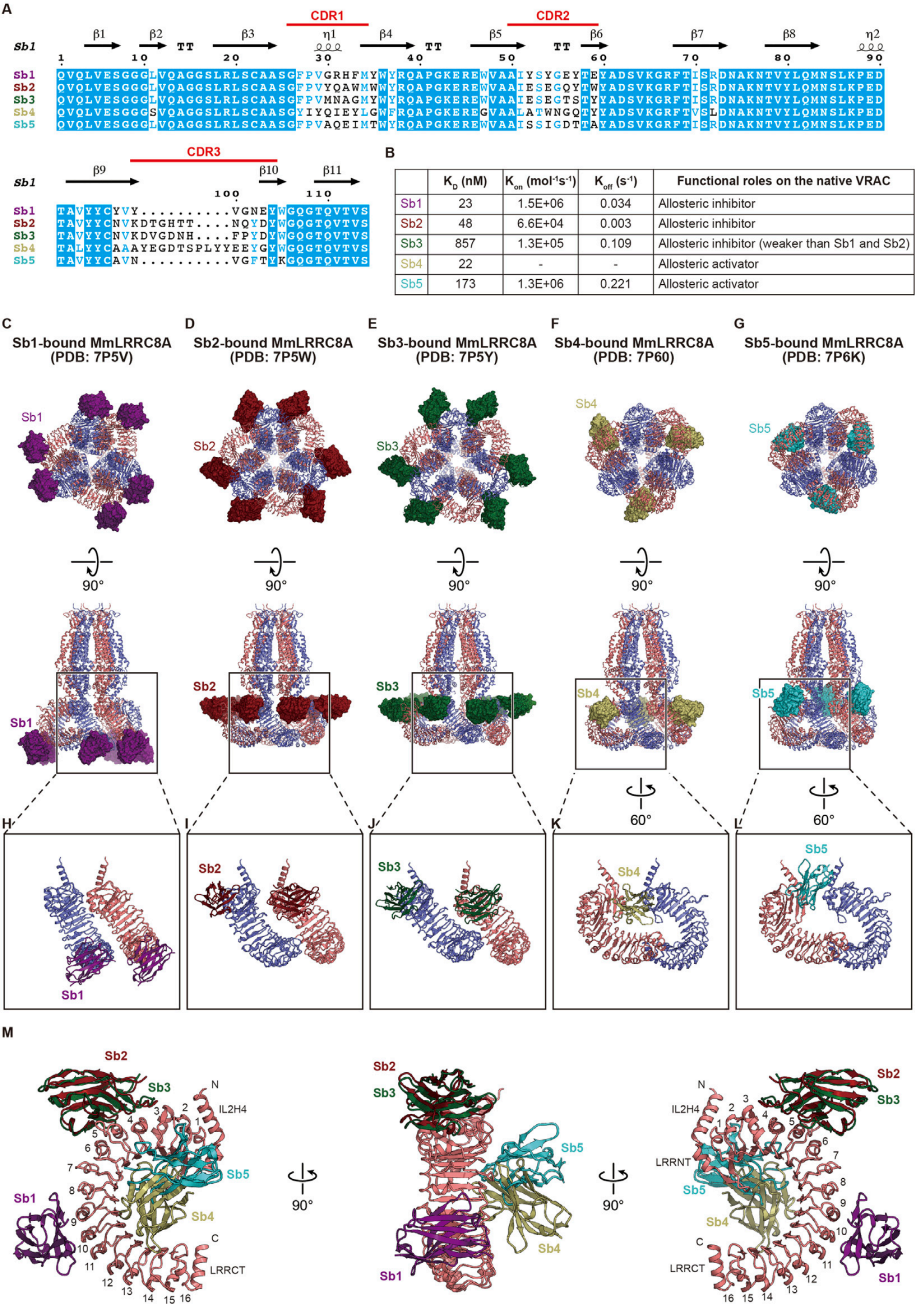
Modulation of the LRR region. **(A)** Sequence alignment of the five sybodies screened by Deneka et al.(Deneka et al., 2021). The secondary structure elements from Sb1 (PDB: 7P5V) (Deneka et al., 2021) and their complementarity-determining regions (CDR) are labeled above the alignments **(B)** Summary of the kinetic and dissociation constants as well as functional roles on the native VRAC current in each sybody. The kinetic and dissociation constants were the average of two independent biological replicates obtained by surface plasmon resonance spectroscopy (SPR). For Sb4, the average K_on_ and K_off_ values were not obtained due to missing data from a replicate. For detailed information, see Table 1 in Deneka et al. (Deneka et al., 2021) **(C–G)** Overall structure of the MmLRRC8A hexamer in complex with each sybody, viewed from the intracellular side (upper) and parallel to the membrane (lower). Each sybody is shown as the surface model and is colored as follows: Sb1, dark purple; Sb2, dark red; Sb3, dark green; Sb4, dark khaki; and Sb5, dark turquoise blue **(H–L)** Close-up views of the interaction between LRR and each sybody. In **(H–J)**, two neighboring LRR domains in complex with each sybody at the tight interface are shown. In **(K,L)** two neighboring LRR domains in complex with each sybody at the loose interface are shown **(M)** Superimposition of each sybody on an LRR domain. Sb2 to Sb5 are superimposed on LRR of the LRR-Sb1 complex using each LRR domain of Sb2 to Sb5. In the right and left panels, LRR numbers are labeled.

Binders that fix target proteins on specific conformations are powerful tools for basic biology and drug discovery. The synthetic single domain antibody (sybody) is a recently developed synthetic antibody that is engineered on the basis of prototypical camelid nanobody structures and has randomized amino-acid residues in all of the three complementarity-determining regions (CDR) (Figure 4A) (Zimmermann et al., 2018). Consequently, the sybody library, which contains about ∼10^12^ sybodies, is an *in vitro* selection platform for specific binders against membrane proteins of interest. Using the sybody library, Deneka et al. (Deneka et al., 2021) identified five sybodies, termed Sb1 to Sb5, that form stable complexes with the LRR region of MmLRRC8A but not with the LRR regions of MmLRRC8C and MmLRRC8D (Figure 4A). When expressed in the cytoplasm of HEK 293 cells, while Sb1, Sb2, and Sb3 inhibited the native VRAC currents, Sb4 and Sb5 increased the currents by about 50% compared to WT. These results demonstrated that, Sb1, Sb2, and Sb3 function as allosteric inhibitors to MmLRRC8A, whreas Sb4 and Sb5 function as allosteric activators (Figure 4B). Deneka et al. (Deneka et al., 2021) determined the structures of the MmLRRC8A homo-hexamer in complex with each sybody (Figures 4C–M). In all of the sybody-bound structures, the overall architecture of MmLRRC8A has three-fold symmetry, being consistent with the structures of MmLRRC8A in detergent (Deneka et al., 2018) and HsLRRC8A in detergent (Kasuya et al., 2018; Kefauver et al., 2018). In contrast, the sybodies showed different binding manners. In the complexes with Sb1, Sb2, and Sb3, each sybody interacts with the individual MmLRRC8A subunit on the convex side of LRR with a stoichiometry of 1:1 LRRC8A:sybody (Figures 4C–E, H–J). Sb1 recognizes the LRR region on repeats 8–11. Sb2 and Sb3 recognize the same epitopes of the LRR region on repeats 3–6, which is closer to the TM region than Sb1 (Figure 4M). In the complexes with Sb4 and Sb5, each sybody interacts with the individual MmLRRC8A subunit on the edge between the flat face and the concave inside of LRR with a stoichiometry of 2:1 LRRC8A:sybody (Figures 4F,G,K,L). Sb4 recognizes the LRR region on repeats 2–14 and Sb5 recognizes the LRR region on repeats 2–7 (Figure 4M). A Förster resonance energy transfer (FRET) study using C-terminally CFP and YFP-fused HsLRRC8A subunits suggested that the VRAC activation induces a conformational change of the LRR region (König et al., 2019). In addition, an electrophysiological study demonstrated that the C-terminally Venus-fused HsLRRC8A and mCherry-fused HsLRRC8E expressed in *Xenopus* oocytes have increased activities, compared to the unfused counterparts (Gaitán-Peñas et al., 2018, Gaitán-Peñas et al., 2016). Considering these observations in FRET and electrophysiological studies, the sybody binding to the LRR region may affect the conformational mobility of the LRR region and thereby alter the channel activity, although further analyses are required to understand the precise effect of each sybody.

## Conclusion and Perspectives

In this review, we summarized the recent advances in structural biology of the LRRC8 family, the pore-forming component of VRAC. Although the summarized structural studies have greatly extended our understanding of the molecular mechanisms of this protein family, there are still various questions to be answered. In terms of biophysical properties of LRRC8, the precise stoichiometry and subunit arrangement are important questions, which may be answered by structural biology methods. Although the stoichiometry of LRRC8 isoforms forming VRAC is known to determine the biophysical properties of VRAC including open probability (Syeda et al., 2016), gating kinetics (Voss et al., 2014; Ullrich et al., 2016), and substrate specificity (Planells-Cases et al., 2015; Gaitán-Peñas et al., 2016; Lutter et al., 2017; Schober et al., 2017; Lahey et al., 2020), the precise stoichiometry and subunit arrangement of LRRC8 isoforms forming the native VRAC are currently unknown. Moreover, previous immunoblotting and efflux studies suggested that the incorporation of only one LRRC8A subunit into a hetero-hexamer is sufficient for the formation of a functional VRAC (Pervaiz et al., 2019) and that a functional VRAC hetero-hexamer may contain three or more LRRC8 isoforms (Lutter et al., 2017). For this question, structure determination of native LRRC8 proteins isolated from animal origins by using specific binders (e.g., Fab, scFv, and Sybody) may be one of the effective approaches, as in the case of the structure determination of native AMPA receptors from the rat brain (Zhao et al., 2019) and mouse brain (Yu et al., 2021) as well as the native glycine receptor from pig spinal cord and brainstem (Zhu and Gouaux, 2021). Another important question is the gating mechanism. For example, while VRAC activity has been shown to be modulated by low intracellular µ (Chen et al., 2019; Strange et al., 2019), a recent FRET study suggested that VRAC activity is not modulated by intracellular µ change but rather by diacylglycerol (DAG)-dependent protein kinase D (PKD) activity in the cellular environment (König et al., 2019). For this discrepancy regarding the gating mechanism, structure determination of LRRC8 embedded in liposomes under the conditions of similar lipid compositions to those found in cell membranes and mimicking cell swelling or cell shrinkage may be one of the effective approaches. The structure determination of a membrane protein embedded in liposomes was achieved in the case of the well-studied multidrug-resistant transporter AcrB (Yao et al., 2020).

In contrast, there are several questions that seem to be difficult for structural biology methods to answer. For example, we previously determined the structure of the HsLRRC8D homo-hexamer (Nakamura et al., 2020) in an attempt to reveal the mechanisms underlying LRRC8D-dependent VRAC activity such as selective transports of uncharged osmolytes (Lutter et al., 2017) and platinum-containing drugs (Planells-Cases et al., 2015). However, it mostly did not go well since the extracellular and intracellular regions, especially the EL1 and IL1 loops, were not resolved in the HsLRRC8D structure (Nakamura et al., 2020). Notably, these loops are highly variable among the LRRC8 isoforms (Abascal and Zardoya, 2012) and a previous electrophysiological study using chimeric homo-hexamer constructs, in which IL1 or EL1 was replaced with the corresponding sequence of other isoforms, suggested that these loops are important for the biophysical properties of VRAC including substrate permeability, rectification, and voltage sensitivity as well as the subunit-subunit interactions (Yamada and Strange, 2018). Considering that the EL1 and IL1 loops are also not resolved in the MmLRRC8A (Deneka et al., 2018) and HsLRRC8A (Kasuya et al., 2018; Kefauver et al., 2018) structures, it seems to be difficult to resolve these loops in detail by structural biology methods.

Overall, considering that the physiological and medical importance of LRRC8 has been gradually revealed in recent years, additional structures and structure-based functional analyses of LRRC8 are necessary to extend our understanding of the molecular mechanisms of this protein family.
